# Acute Chagas disease in Amazonia, western Pará: perspectives from medical assistance to genetic elucidation

**DOI:** 10.1590/0074-02760240108

**Published:** 2025-04-25

**Authors:** Helena Rangel Esper, Vera Lúcia Teixeira de Freitas, João Guilherme Pontes Lima Assy, Olívia Campos Pinheiro Berreta, Alisson dos Santos Brandão, Erika Yoshie Shimoda Nakanishi, Claudia de Abreu Fonseca, Francisco Oscar de Siqueira França, Marta Heloísa Lopes

**Affiliations:** 1 Universidade de São Paulo, Faculdade de Medicina, Departamento de Infectologia e Medicina Tropical, Núcleo de Medicina Tropical, São Paulo, SP, Brasil; 2 Universidade de São Paulo, Faculdade de Medicina, Departamento de Infectologia e Medicina Tropical, São Paulo, SP, Brasil; 3 Secretaria Municipal de Saúde de Santarém, Hospital Municipal de Santarém, Santarém, PA, Brasil; 4 Universidade de São Paulo, Faculdade de Medicina, Hospital das Clínicas, Laboratório de Investigação Médica em Imunologia, São Paulo, SP, Brasil

**Keywords:** Chagas disease, Amazonian ecosystem, infectious disease transmission, vertical, Trypanosoma cruzi, molecular characterization, discrete typing units

## Abstract

**BACKGROUND:**

The experience of the USP Tropical Medicine Centre (NUMETROP) team in providing medical care during acute Chagas disease (ACD) outbreaks in Santarém, Pará, motivated this study.

**OBJECTIVES:**

To study the epidemiological, clinical-laboratory, and socio-cultural aspects of confirmed cases of ACD in outbreaks in Santarém from March 2016 to March 2018.

**METHODS:**

Observational case series study of ACD outbreaks in two communities: Marimarituba in 2016 and Cachoeira do Aruã in 2017. Diagnostic characterisation included classification into discrete typing units (DTUs).

**FINDINGS:**

Eight cases were diagnosed as ACD TcIV in Marimarituba and seven cases were identified as ACD TcI in Cachoeira do Aruã. Women of childbearing age were numerous in both groups, and one miscarriage and two possible vertical transmissions were observed. Fever and rash were the most common findings in Marimarituba, with a fatality rate of 12.5%. In both outbreaks, serological surveillance was performed three to 21 months after treatment, with no confirmation of a “serological cure”.

**MAIN CONCLUSIONS:**

We observed possible vertical transmission, diverse DTUs in the same municipality, and a lack of knowledge about patient outcomes. We highlight that, despite the importance of ACD in the Amazon region, there is no institutional follow-up of patients from diagnosis to cure.

Chagas disease (CD) is an enzootic disease primarily affecting wild and domestic animals. It started being transmitted to humans accidentally by invading and exploiting wild habitats.^1^ The first CD cases diagnosed in the Amazon region occurred in French Guiana (1941) and in the Brazilian Amazon (1968).^2^ The disease is complex and heterogeneous, progressing from the acute to the chronic phase and presenting a wide range of symptoms during its natural history. The diversity of clinical manifestations makes diagnosis and identification difficult in the medical field.^1^ ^,^ ^3^

Multiple routes of infection with *Trypanosoma cruzi* and acute Chagas disease (ACD) outbreaks due to oral transmission reported in the Amazon are causes for concern. Considering biological and socio-cultural factors, it is crucial to have a deeper understanding of the disease. A comprehensive understanding of ACD could help prevent the emergence of this disease due to ecological imbalances and inform more effective management strategies for future outbreaks.

Oral transmission occurs mainly through food contaminated with faeces or maceration of the infected vector; the different modes of transmission present differences in the number of parasites inoculated at the time of infection, leading to variations in incubation time, number of circulating parasites, pathogenicity, prognosis and even the accuracy of different diagnostic tests.^4^ ^,^ ^5^ ^,^ ^6^ ^,^ ^7^ ^,^ ^8^

*Trypanosoma cruzi* exhibits significant diversity and is currently classified into six discrete typing units (DTUs) labelled TcI through TcVI, along with an additional DTU known as TcBat. These DTUs are found in *T. cruzi* transmission cycles across various ecotopes and exhibit distinct epidemiological, biological, and clinical characteristics that remain only partially understood. The DTU TcI group is the most frequently identified, mainly in Central America. In Brazil, DTU TcI predominates in 66% of human isolates. TcIV DTU has been described in Bolivia, Venezuela, Brazil, Peru, and Colombia, accounting for 3.6% of identifications.^9^

The Brazilian Ministry of Health recorded 112 outbreaks of ACD in the national territory between 2005 and 2013, exclusively in the Amazon region, with an increase in notifications from 2007 to 2020.^10^ Most of the outbreaks, 75.9%, occurred in the State of Pará, and the probable source of infection was the consumption of food contaminated with *T. cruzi*, açaí (*Euterpe oleracea*), bacaba (*Oenocarpus bacaba*). Currently, oral transmission of CD is recognised as the main mode of transmission of this neglected disease in the Amazon basin.^11^

The description of the clinical and laboratory characteristics of patients with ACD from two different outbreaks in western Pará, together with the characterisation of their different DTUs, provides new perspectives on the presentation of this disease. We also highlight the difficulties encountered in patient care.

## SUBJECTS AND METHODS

*Population* - Observational study characterising confirmed cases from two ACD outbreaks that occurred in March 2016 and August 2017, in the riverine communities of Marimarituba (2º12’29”S 55º1’14”W) and Cachoeira do Aruã (2º38’58”S 55º43’24”W). Both are located in the municipality of Santarém, State of Pará, northern region of Brazil. Marimarituba is located on the banks of the Amazon River, on an island about 60 km from the centre of Santarém. Cachoeira do Aruã is located on a tributary of the Tapajós River, 105 km from the centre of Santarém. Both can only be reached by the river, a 3 h and day trip by motorboat, respectively.

Cases from Marimarituba were identified and diagnosed in March 2016 by infectious diseases specialists (IDS) associated with the Tropical Medicine Centre (NUMETROP) of the Department of Infectious and Parasitic Diseases, Faculty of Medicine, University of São Paulo. Fifteen patients diagnosed with ACD were followed at the Hospital Municipal de Santarém (HMS) and the Infectious Diseases Outpatient Clinic of the Municipality of Santarém (IDOC). The index case from Marimarituba was referred for IDS evaluation after *T. cruzi* trypomastigotes were observed in the microscopic analysis of the complete blood count. The cases from Cachoeira do Aruã were referred to IDOC by the *in loco* state surveillance expedition in September 2017.

Data collection from confirmed individuals was standardised using a questionnaire. Cases were considered confirmed on the basis of a positive smear or serology together with a suggestive clinical presentation and an epidemiological link to ingestion of suspected juice, as recommended by the National Surveillance Guide.^12^ The median time from suspicion to serological confirmation and feedback to patients was 60 days. All confirmed patients were treated with benzonidazole (BZ) and followed up with NUMETROP.

*Sample collection* - Blood samples were collected in the municipality of Santarém, State of Pará. Smear and serology: indirect haemagglutination assay (IHA), enzyme-linked immunosorbent assay (ELISA) and indirect immunofluorescence (IIF) were performed at HMS and Laboratório Central do Estado do Pará (LACEN-PA). Other samples were sent to the Laboratory of Medical Research in Immunology, Hospital das Clínicas, Faculty of Medicine, University of São Paulo, where the characterisation of *T. cruzi* was performed.

*Ethics* - The study was submitted to and approved by the Ethical Review Committee of the Faculty of Medicine of the University of São Paulo (approval number 2.728.843). The procedures were carried out in accordance with the ethical standards of the committee. Patients diagnosed with CD were treated according to the guidelines of the Brazilian Ministry of Health.

## RESULTS

Eight cases were diagnosed as ACD TcIV in Marimarituba and seven as ACD TcI in Cachoeira do Aruã.^13^

*Sociodemographic and clinical characteristics of ACD cases* - The intrafamilial outbreaks occurred after consumption of artisanal beverages contaminated with *T. cruzi*, in this case bacaba wine (*Oenocarpus bacaba*) in Marimarituba and pataua juice (*Oenocarpus bataua*) in Cachoeira do Aruã. Therefore, the route of transmission was oral for the eight inhabitants of Marimarituba and the seven from Cachoeira do Aruã. There was a predominance of females, with 62.5% in the Marimarituba outbreak and 57.1% in Cachoeira do Aruã, four out of nine were of childbearing age. The mean age was similar between groups, 23 years in Cachoeira do Aruã and 26 years in Marimarituba. The mean incubation period was four days in the Cachoeira do Aruã cases and 15 days in the Marimarituba cases.

The signs and symptoms observed in both outbreaks are listed in Table I.

**TABLE I:** Frequency of symptoms in acute Chagas disease (ACD) cases in Marimarituba’s and Cachoeira do Aruã’s outbreak in 2016 and 2017

Signs and symptoms	Marimarituba	Aruã
	% (N = 8)	% (N = 7)
Fever	100.0	100.0
Headache	100.0	100.0
Myalgia	75.0	57.1
Edema	100.0	71.4
Gastrointestinal symptoms	62.5	28.6
Arthralgia	62.5	14.3
Rash	75.0	14.3
Adenopathy	50.0	NA
Nervous System alterations	12.5	NA
Cardiovascular symptoms	12.5	14.3
Hemorrhagic manifestestations	0.0	14.3
Respiratory symptoms	0.0	14.3

N: number; NA: not available.

*Diagnostic laboratory* - Direct microscopic examination of peripheral blood showed a positivity rate of 87.5% in the Marimarituba outbreak and 100% in the Cachoeira do Aruã outbreak.

Considering the IgM and IgG IIF and IHA methods, serology was positive in 100% of the Marimarituba ACD cases, but no reactive result was observed in the ELISA test in any of the cases. The infant with possible congenital transmission was diagnosed by direct microscopic examination. In the Cachoeira de Aruã cases, IgM and IgG IIF, IHA and ELISA serological tests were performed mainly after treatment, with IgM IIF positivity in 14% and IgG IIG positivity in 43%.

Qualitative polymerase chain reaction (PCR) positivity was 87.5% in the Marimarituba outbreak and 85.7% in the Cachoeira do Aruã outbreak, and the quantitative test was positive in 62.5% of the Marimarituba cases and 85.7% of the Cachoeira do Aruã cases.

*Treatment and outcomes* - The lethality of the Marimarituba outbreak was 12.5%; the fatal index case was a child aged 1 year and 10 months who had started treatment and remained in hospital for only 10 days. We have published an article discussing the possible factors influencing this outcome.^14^

In total, 16 patients were treated with BZ for 60 days; one case turned out to be a false-positive smear and was treated unnecessarily. No serious adverse drug reactions were observed, and no “serological cure” was confirmed at a maximum follow-up of 21 months. There were two possible vertical transmissions, one in each outbreak, and one miscarriage, which we could not prove to be related to ACD. The first possible vertical transmission of DTU TcIV was published by our group.^15^ The detection of DTUs in the outbreaks, the methodology and the discussion of the results have already been disclosed to the scientific community.^13^

In the Marimarituba outbreak, the first follow-up sample was collected from six patients and subjected to serological testing with ELISA IgG and IHA eight months after treatment, with negative results in all patients. The only serological sample collected from the infant showed an indeterminate result in the ELISA IgG assay four months after cessation of treatment (Table II).

**TABLE II:** Serological monitoring of patients from Marimarituba before and after treatment

Age (Y) /Sex M or F	Pre-treatment T0		Post-treatment T1		Month	Post-treatment T2		Month
	Result	Serology	Result	Serology		Result	Serology	
18/F	Positive	IIF IgM 1/160; IIF IgG 1/320; ELISA IgG NR; IHA R	Negative	ELISA IgG and IHA NR	8	ND	NA	NA
16/M	Positive	IIF IgM 1/160; IIF IgG 1/160; ELISA IgG NR; IHA R	Negative	ELISA IgG and IHA NR	8	ND	NA	NA
20/M	Positive	IIF IgM 1/160; IIF IgG 1/320; ELISA IgG NR; IHA R	Negative	ELISA IgG and IHA NR	8	ND	NA	NA
56/M	Positive	IIF IgM 1/80; IIF IgG 1/160; ELISA IgG NR; IHA R	Negative	ELISA IgG and IHA NR	8	ND	NA	NA
31/F	Positive	IIF IgM 1/320; IIF IgG 1/640, ELISA IgG NR; IHA R	Positive	IIF IgG 1/80; IHA R	4	Positive	IIF IgG 1/320; ELISA IgG and IHA NR	11
1/F †	Positive	IIF IgM 1/80; IIF IgG 1/160; ELISA IgG NR; IHA R	NA	NA	NA	ND	NA	NA
6/F	Positive	IIF IgM 1/80; IIF IgG 1/640; ELISA IgG NR; IHA R	Negative	ELISA IgG and IHA NR	8	Positive	IIF IgG 1/80; ELISA IgG and IHA NR	17
55/F	Positive	IIF IgM 1/80; IIF IgG 1/80; ELISA IgG NR; IHA R	Negative	ELISA IgG and IHA NR	8	Inconclusive	IIF IgG 1/40; ELISA IgG NR; IHA NR	15
0/F*	ND	NA	Inconclusive	ELISA IgG Ind; IHA NR	4	ND	NA	NA

Y: years; M: male; F: female; IIF: indirect imunofluorescence assay; ELISA: enzyme-linked immunosorbent assay; IHA: indirect haemagglutination assay; NR: no reagent; R: reagent; ND: not done; NA: not applicable; Ind: indeterminate; †: death; ^*^infant, congenital?

In the same outbreak, a patient who was four weeks pregnant started treatment after a miscarriage that occurred at 15 weeks’ gestation. The patient voluntarily discontinued treatment after 23 days and treatment was restarted after 54 days, this time with good compliance, according to the patient. The IIF test result was positive 11 months after the first treatment attempt, at which time she was again six weeks pregnant. (Table II, Figure).

**Figure:**
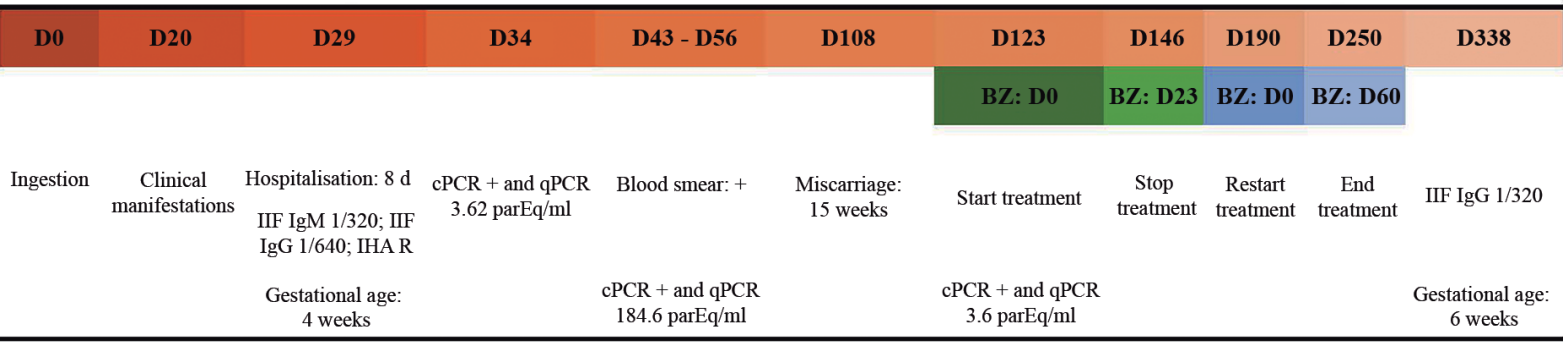
Follow-up of a pregnant patient from Marimarituba with acute Chagas disease (ACD) after consumption of bacaba wine.

Serological monitoring was performed at four and 11 months after BZ therapy, and there was no confirmation of “serological cure” (Table II).

The seven patients from Cachoeira de Aruã were also treated with BZ, including the infant who was diagnosed by conventional PCR (cPCR) and quantitative PCR (qPCR), the latter showing 78.3 par/mL blood.

Samples taken three months after the end of treatment were negative in three patients and inconclusive in two others (Table III).

**TABLE III:** Serological monitoring of patients from Cachoeira do Aruã before and after treatment

Age (Y)/ Sex M or F	Pre-treatment T0		Post-treatment T1		Month
	Result	Serology	Result	Serology	
18/F	Positive	IIF IgM 1/160; IIF IgG 1/1280; ELISA IgG e IHA R	Positive	IIF IgG 1/80; IHA R	21
14/M	ND	NA	Inconclusive	IIF IgG NR, ELISA IgG Ind; IHA NR	3
48/M	ND	NA	Negative	IIF IgG; ELISA IgG and IHA NR	3
48/F	ND	NA	Inconclusive	IIF IgG 1/40; ELISA IgG and IHA NR	3
18/F	ND	NA	Positive	IIF IgG 1/80; ELISA IgG and IHA NR	3
9/F	ND	NA	Negative	IIF IgG, ELISA IgG and IHA NR	3
4/M	ND	NA	Negative	IIF IgG, ELISA IgG and IHA NR	3
0/F^*^	ND	NA	Positive	IIF IgG NR; IHA R	21

Y: years; M: male; F: female; IIF: indirect imunofluorescence assay; ELISA: enzyme-linked immunosorbent assay; IHA: indirect haemagglutination assay; NR: no reagent; R: reagent; ND: not done; NA: not applicable; Ind: indeterminate; ^*^infant, congenital?

Three patients remained seropositive three to 21 months after treatment. Two were women of childbearing age and the other was the child of one of them. A sample from the infant taken 21 months after treatment was still positive for HAI. There were symptomatic family members of the Marimarituba outbreak who did not have serology repeated to exclude infection because of lack of follow-up. It is uncertain whether they consumed the same batch of bacaba as the confirmed cases.

Marimarituba and Cachoeira do Aruã are only accessible by river, 3 h and a day by motorboat respectively. There is no guarantee of local support for people who may be infected. In addition, there is no funding available for people who need to travel to the city centre for fever symptoms or follow-up care. Instead, patients are expected to pay for their own transportation. In our experience, families from Marimarituba and Cachoeira do Aruã have attended more than ninety outpatient appointments using their own resources after liaison with the NUMETROP team.

## DISCUSSION

This paper analyses the clinical-laboratory aspects of outbreaks of different DTUs. At present, it is not possible to conclude that there are different clinical presentations of ACD for each DTU. However, it is necessary to explore possible correlations based on the results found.

The different incubation period in the reported ACD outbreaks, 15 days for the confirmed group from Marimarituba and four days for the one from Cachoeira do Aruã, is an aspect that deserves attention. The possible factors involved are inoculum and parasite load, variations in distribution between tissues, tissue invasion, parasite replication, host immune response and the genetic difference between *T. cruzi*.^2^ ^,^ ^16^ An incubation period of four days on average in an ACD outbreak, as occurred in Cachoeira do Aruã, is within the period calculated and published by PAHO.^17^ This short incubation period may therefore be due to the oral route, with a high parasite load, comorbidities that affect the immune response, such as anaemia due to malnutrition, or even TcI DTU, which may have greater tissue dissemination.^18^

Although not applicable in routine clinical practice, the molecular PCR techniques and the elucidation of the DTU carried out in this study have triggered relevant discussions on the characteristics of *T. cruzi* in humans and in its ecotope. Beyond the clinical and genetic perspectives, it is important to look at the social and cultural aspects of these outbreaks, as they represent some of the challenges we faced and address some key issues in the management of ACD in the Amazon.

In the Patacho region, where the people of Marimarituba collect bacaba, there was an increase in deforestation one year before the outbreak of ACD caused by TcIV DTU. As stated by Valente et al., the eco-epidemiological relationships between reservoirs, vectors and ecotopes in the transmission cycle of the TcIV strain are unclear,^2^ as is its relationship with anthropogenic interventions in landscapes.

In this study, the DTU Tc IV outbreak had a higher incidence of lymphadenopathy, rash, gastrointestinal symptoms, arthralgia, and there was greater lethality in relation to the TcI outbreak. Of note, these signs and symptoms suggest a greater activation of the mononuclear phagocytic system and a greater association with hypersensitivity, therefore an exacerbation of the immune response. These factors may correlate with lower tolerance to the parasite, less control of parasitaemia and tissue dissemination. In contrast to TcIV, there is experimental evidence in mice that shows greater tolerance to acute TcI infection,^18^ possible adaptation of DTU to parasitism, which may be related to the fact that the lineage is considered ancestral.^19^ This “adaptation” would possibly lead to an outbreak with less exuberance of symptoms, lower lethality and possibly a shorter incubation period, as observed in Cachoeira do Aruã.

Santarém is a large municipality with an area of 17,898.389 km², mostly forested, with only 97 km² in the urban perimeter. Different DTUs circulate in different wild mammals and vectors in the region. Among the DTUs, TcI is the one that is widely distributed throughout the Americas and occurs ubiquitously in the sylvatic cycle.^19^ ^,^ ^20^ Human infection with TcI has been described mainly in northern South America, Central America and Mexico. DTU TcIV, like TcI, is associated with the wildlife cycle and is found in North American raccoons, South American primates and coatis.^5^ There have also been reports of oral outbreaks in the Brazilian Amazon.^21^

From a demographic perspective, part of the population identifies as indigenous, descendants of the Arapium and Jaraqui peoples. The reported outbreaks of ACD in the two groups studied were predominantly female. Most reports do not describe or discuss the impact of female involvement in outbreaks of oral transmission; identifying and treating infected women of childbearing age who are not pregnant may be an effective strategy to reduce vertical transmission to future offspring.

There was also one abortion and two cases of vertical transmission probably related to *T. cruzi* infection; better knowledge of the route and timing of maternal-foetal infection are factors that can play an important role in controlling transmission; both were vaginal births. One of the difficulties in caring for children exposed to *T. cruzi* is loss of follow-up, as occurred in the cases in this study. A prenatal screening programme successfully implemented in Mato Grosso do Sul recommends including *T. cruzi* serology in addition to syphilis and HIV testing.^22^ This integration would potentially reduce the vulnerability of pregnant women, foetuses, mothers and children exposed to *T. cruzi* and diagnosed with ACD.

The prevalence of mother-to-child transmission of CD in the northern region of Brazil remains poorly understood. A systematic review and meta-analysis of the prevalence of congenital transmission in Brazil, published in 2014, did not include epidemiological data from the northern region, despite this being the region with the highest number of reported ACD cases in Brazil.^22^

Regarding cultural aspects, the concept that food habits and practices can transmit *T. cruzi* is a remarkable challenge for the rural riverine communities affected by ACD, as it has only recently been concluded. On the other hand, the technical conditions for harvesting, pulping and consumption have been known for more than a century,^23^ as the daily relationship of the northern riverine culture with food coming directly from rivers and forests, through fishing, hunting and fruit gathering.^24^ Attempts to introduce bleaching, a technique developed to inactivate the parasite in palm fruit juice and promoted by government policies, have not been fully supported by riverine communities, highlighting the difficulty of changing the routine of making these juices.^25^ Eating habits are linked to components of socio-cultural constructions and, above all, to identifications. In this sense, the recognition of foods that may lead to ACD as taboos also depends on the socio-political relations that construct the historical process.

From an epidemiological point of view, the investigation and management of DCA outbreaks can involve many suspected cases and require urgent attention from the political authorities. It requires skilled health professionals, transport for *in situ* investigation and equipment at a critical time. Funding for these activities is not guaranteed, and the uncertainty of the expedition to the riverine communities indicates a programmatic vulnerability. Municipalities have ongoing priorities and reallocation of funds for DCA outbreak management may be discouraged due to budget constraints and political timelines.

Given the specificities of the Amazon, in terms of long distances and hydrographic networks, ethnic, biological and cultural diversity, and the challenges mentioned above, ACD support cannot be separated from the Sistema Único de Saúde (SUS). Integration through the Family Health Programme has been implemented in several states of the country,^26^ which would make longitudinal care more feasible in the Amazonian context. An established and continuous care model can understand and learn how the population relates to ACD through interpretive processes that understand the meaning of the illness. However, without a national medical assistance programme to support it, it is unlikely that this will happen, as other diseases are prioritised at the municipal level in the Amazon.

This study shows some characteristics of the disease in western Pará, such as its lethality, the presence of possible vertical transmission, the diversity of DTUs in the same municipality and the lack of knowledge about patient outcomes, even with a two-year follow-up. It is important to highlight the inextricable link with social and cultural factors and the lack of an institutional SUS programme to follow patients from diagnosis to cure, despite the importance of the disease in the Amazon. We must also mention the difficulty of reconciling prevention without interfering with the food and cultural practices of the region. It is essential to point out these aspects, which have influenced the outbreaks and which are little remembered.
